# Response to: The difference in the association between included ECPR patients and neurological outcomes

**DOI:** 10.1186/s13054-023-04342-8

**Published:** 2023-02-10

**Authors:** Makoto Watanabe, Tasuku Matsuyama, Tetsuhisa Kitamura

**Affiliations:** 1 Department of Emergency Medicine, Kyoto Prefectural University of Medicine, Kamigyo-ku, Kyoto 602-8566 Japan; 2 Division of Environmental Medicine and Population Sciences, Department of Social and Environmental Medicine, Graduate School of Medicine, Osaka University, 2-2 Yamadaoka, Suita, Osaka 565-0871 Japan

## Dear editor,

We express our gratitude to Hifumi and their associates for their interest in our manuscript pertaining to the utilization of targeted temperature management (TTM) among patients treated with extracorporeal membrane oxygenation (ECMO) [1].

As noted, the optimal target temperature may vary contingent upon the aetiology of arrest. While 91% of our study cohort had a cardiac cause of arrest, the impact of the remaining patients on the outcome of our study may be non-negligible. Therefore, we conducted a sensitivity analysis that included only the patients with a cardiac cause of arrest and found the results to be consistent (see Table 1).

**Table 1:** Sensitivity analysis (including only the patients with a cardiac cause of arrest)

	All patients	Original cohort		Crude analysis OR (95% CI)	Multivariable analysis AOR (95% CI)^a^	Propensity score analysis OR (95% CI)^b^
		n-TTM	h-TTM			
	*N* = 814	*N* = 220	*N* = 594			
30-day neurological favourable outcome	137 (16.9)	38 (17.3)	99 (16.7)	0.96 (0.64–1.44)	0.97 (0.60–1.55)	1.01 (0.65–1.56)
30-day survival	297 (36.5)	81 (36.8)	216 (36.4)	0.98 (0.71–1.35)	0.98 (0.68–1.41)	1.04 (0.74–1.46)

Values are expressed numbers (percentages) unless indicated otherwise

*TTM* Targeted temperature management, *n-TTM* Normothermic TTM, *h-TTM* Hypothermic TTM, *OR* Odds ratio, *AOR* Adjusted odds ratio, *CI* Confidence interval, *IPW* Inverse probability weighting

^a^Shown is the AOR from the multivariable logistic regression analysis adjusted for age, sex, bystander witness, bystander CPR, use of public-access AEDs, prehospital epinephrine administration, prehospital advanced airway management, time from EMS call to contact with the patients, time from EMS contact with the patients to hospital arrival, type of centre, the annual volume of ECMO used for OHCA at each centre, success of PCI, timing of ECMO start, and time from hospital arrival to induction of ECMO

^b^Shown is the odds ratio from the univariable logistic regression analysis with IPW

We concur that there may be an inherent bias, specifically “physician’s discretion”. As Hifumi and colleagues have astutely pointed out, critically ill patients may tend to be treated with higher targeted temperatures. However, we contend that this study possesses a certain degree of robustness as we adjusted for numerous pre- and in-hospital factors that physicians commonly consider when determining target temperature, such as the cause of cardiac arrest, initial documented rhythm at the scene and upon hospital arrival, and time-related variables. Nevertheless, we agree that validation of our findings necessitates an evaluation of the Study of Advanced Life Support for Ventricular Fibrillation with Extracorporeal Circulation in Japan (SAVE-J II) trial, a comprehensive observational study on the same topic that utilizes detailed treatment-related data [2], as well as a meta-analysis. Furthermore, a randomized controlled trial is imperative to determine the optimal target temperature for patients undergoing ECMO.

## Data Availability

Please contact the authors for data requests.
